# L-Arginine and SDMA Serum Concentrations Are Associated with Subclinical Atherosclerosis in the Study of Health in Pomerania (SHIP)

**DOI:** 10.1371/journal.pone.0131293

**Published:** 2015-06-22

**Authors:** Martin Bahls, Nele Friedrich, Dorothee Atzler, Stephan B. Felix, Matthias A. Nauck, Rainer H. Böger, Henry Völzke, Edzard Schwedhelm, Marcus Dörr

**Affiliations:** 1 University Medicine Greifswald, Department of Internal Medicine B, Greifswald, Germany; 2 DZHK—German Centre for Cardiovascular Research, partner site Greifswald, Greifswald, Germany; 3 Institute of Clinical Chemistry and Laboratory Medicine, University of Greifswald, Greifswald, Germany; 4 Division of Cardiovascular Medicine, Radcliffe Department of Medicine, University of Oxford, Oxford, United Kingdom; 5 Institute of Clinical Pharmacology and Toxicology, University Medical Center Hamburg-Eppendorf, Hamburg, Germany; 6 DZHK—German Centre for Cardiovascular Research, partner site Hamburg/Kiel/Lübeck, Hamburg/Kiel/Lübeck, Germany; 7 Institute for Community Medicine, University of Greifswald, Greifswald, Germany; INRCA, ITALY

## Abstract

**Objective:**

Even though ˪-arginine (ARG) derivatives can predict cardiovascular mortality, their role as atherosclerotic biomarkers is unclear. We tested the hypothesis if asymmetric dimethylarginine (ADMA), symmetric dimethylarginine (SDMA) and the sum of both (DMA) are positively, while ARG and ARG/ADMA ratio are inversely associated with carotid intima-media thickness (cIMT) and atherosclerotic plaque in the carotid artery.

**Approach and Results:**

Cross-sectional data of 1999 subjects (age: 45–81 years; 48.2% ♀) from the population-based Study of Health in Pomerania (SHIP-0) was used. Analysis of variance and logistic regression models were calculated and all adjusted models were corrected for sex, age, smoking status, waist-to-hip ratio and estimated glomerular filtration rate. Increased cIMT (>75^th^ age-sex specific percentile) was found in 517 subjects (25.7%), while atherosclerotic plaque was detected in 1413 subjects (70.4%). SDMA tertiles were significantly positively associated with larger cIMT among subjects with high SDMA levels [>66^th^: 0.82 (95%-CI 0.80; 0.85) mm]. High SDMA levels were related to a higher odds ratio (OR) of increased cIMT [OR 1.39 (95%-CI 1.08; 1.79)]. Furthermore, ARG was positively associated with atherosclerotic plaques [OR 1.41 (95%-CI 1.07; 1.85)]. No relation was found for ADMA and atherosclerosis.

**Conclusions:**

In conclusion, the hypothesis of a positive association between SDMA with an increased cIMT was confirmed. Unexpectedly, ARG was positively related to atherosclerotic plaque. In view of these inconsistent findings, the impact of ARG derivatives as atherosclerotic biomarkers deserves further research.

## Introduction

Nitric oxide (NO) is a key signaling molecule for several physiological functions. For example, NO is essential for vascular health by mediating vascular homeostasis, acting antithrombotic and anti-inflammatory [1]. In endothelial cells NO is synthesized from ˪-arginine (ARG) via endothelial NO synthase (eNOS) [2]. While the importance of eNOS for endothelial NO bioavailability has been previously established [3], the significance of ARG is exemplified by the fact that increased exogenous ARG upregulates endothelial NO production [4]. In a clinical setting the infusion of ARG in forearm resistance vessels improved vasodilatory capacity in hypercholesterolemic patients [5]. In addition, the endothelial vasodilatory response was enhanced in patients with atherosclerotic plaques in the left anterior descending coronary arteries after intracoronary ARG infusion [6]. Hence, previous research indicates that increased presence of ARG in the circulation may improve endothelial function and contribute to delayed development of atherosclerosis.

Symmetric and asymmetric dimethylarginine (SDMA and ADMA, respectively) are the products of posttranslational modifications to ARG residues [7]. Recently, plasma concentrations of SDMA and ADMA have been identified as potential biomarkers for cardiovascular disease (CVD). Specifically, circulating levels of ADMA and SDMA were positively associated with all-cause and cardiovascular mortality in patients with stable coronary heart disease [8], peripheral arterial disease [9], and end-stage renal disease [7]. From a pathophysiological point of view both derivatives may contribute to the early stages of atherosclerosis. SDMA prevents cellular ARG uptake and ADMA inhibits eNOS, thereby inducing endothelial dysfunction via a reduction in NO bioavailability [10]. Furthermore, ADMA has been associated with several cardiovascular risk factors and advanced atherosclerotic disease progression [9,11,12]. In addition, SDMA has been associated with subclinical atherosclerosis in an elderly population [13]. Hence, circulating ADMA and SDMA concentrations are potential biomarkers for atherosclerotic disease progression. While the predictive value of ARG derivatives as atherosclerotic disease biomarkers in patient populations have been shown previously [7–9], their role as predictors of atherosclerosis in population-based cohorts is currently uncertain.

Due to the paucity of literature discussing the relation between ARG derivatives and atherosclerosis in population-based cohorts, the aim of this study was to provide information for this knowledge gap. We used carotid intima-media thickness (cIMT) and presence of atherosclerotic plaques in the extracranial common carotid artery as diverse atherosclerotic prognostic markers. While cIMT is a well-known indicator of early atherosclerosis and a predictor of advanced disease [14], the presence of atherosclerotic plaques is related to adverse cardiovascular outcomes [15]. To investigate if ARG derivatives are linked with atherosclerosis, we associated serum concentrations of ARG, ADMA and SDMA as well as the sum of the dimethylarginines (DMA; ADMA + SDMA) and the ARG/ADMA ratio with cIMT and the presence of atherosclerotic plaque in a large sample form of an adult population-based cohort.

## Materials and Methods

### Study population

The presented data were derived from a population-based cohort in West Pomerania in Germany [16]. Recruitment strategy and study design have been reported elsewhere [17]. Briefly, between 1997 and 2001 a total of 6265 subjects (age 20 to 79) were invited to participate in the study. At baseline 4308 men and women agreed to participate in the comprehensive examination program (response = 68.8%) [17]. The study was approved by the ethics committee of the University of Greifswald, complies with the Declaration of Helsinki and all study participants gave written informed consent. SHIP data are publically available for scientific and quality control purposes. Data usage can be applied for via www.community-medicine.de.

Carotid IMT was assessed in all subjects aged 45 or older (n = 2578). For the present analysis subjects with severely impaired renal function [estimated glomerular filtration rate (eGFR) < 30 mL/min/1.73 m^2^] were excluded (n = 2560). After exclusion of subjects without available ARG derivative concentrations, 1999 remained in the sample.

### Interview, medical and laboratory examination

Trained and certified staff used standardized computer-assisted interviews to ask the patients about their age, sex, smoking habits, and physical activity behavior. Smoking habits were classified as current smoker, nonsmoker or former smoker. Being physically active was defined as at least one hour per week of leisure time exercise. In addition, waist circumference (WC) was assessed to the nearest 0.1 cm using an inelastic tape measure. The subject was standing comfortably with body weight evenly distributed between both feet. WC was measured midway between the lower rib margin and the iliac crest in the horizontal plane. Waist-to-hip ratio (WHR) was calculated as the WC (in cm) divided by hip circumference (in cm). Diabetic patients were identified based on the self-reported use of antidiabetic medication [anatomic, therapeutic, and chemical (ATC) code: A10] in the last 7 days or a glycosylated hemoglobin level > 6.5%. Blood pressure (BP) was assessed after a 5 min resting period in sitting position. Systolic and diastolic BP were measured three times, with three minutes rest in between, on the right arm using a digital blood pressure monitor (HEM-705CP, Omron Corporation, Tokyo, Japan). The average of the second and third reading was used. Hypertensive patients were identified by either self-reported antihypertensive medication or a systolic BP above 140 mmHg and/or a diastolic value of more than 90 mmHg.

A non-fasting venous blood sample was drawn from all subjects in supine position (between 7 am and 4 pm). The eGFR was calculated according to Stevens et al. [18] [eGFR = 186 x (plasma creatinine concentration x 0.0113118)^-1.154^ x age^-0.203^) multiplied by 0.742 for female subjects] and expressed as mL/min/1.73 m^2^. Established and validated protocols for liquid chromatography-tandem mass spectroscopy (LC-MS/MS) were used to assess serum ARG and ADMA and SDMA concentrations [19]. Briefly, 25 μL of serum were diluted in methanol with stable isotope labeled ARG, ADMA, and SDMA. Thereafter, the guanidine compounds were converted into their butyl esters. Guanidino compound concentrations were calculated using triplicates with calibration curves based on four levels. Platewide quality controls (QC) were run in two levels by duplicates. A second analysis was done on the samples to assess coefficient of variation and bias of QC, which had to be below 15%. The ARG/ADMA ratio and sum of the DMA (ADMA + SDMA) was calculated.

### Ultrasound measurements

The ultrasound protocol employed to measure cIMT and evaluate the presence of atherosclerotic plaque has been described previously [20]. Briefly, left and right extracranial carotid arteries were scanned bilaterally with a B-mode ultrasound using a 5-MHz linear array transducer and high-resolution instrument (Diasonics VST, Gateway, Santa Clara, CA, USA). Ultrasound images from the distal straight portion before the bifurcation (1 cm in length) were recorded. Far-wall cIMT was calculated by averaging 10 consecutive measurement points with 1 mm in between from the bulb of both sides. Carotid IMT was defined as the averaged maximal IMT measurement from the left and right extracranial carotid arteries. In 115 subjects, missing data occurred for cIMT measurements by exclusion criteria (e.g. bandages, dressings, scars, vessel kinking, or ultrasound image quality too poor). After exclusion of missing data, a total of 1999 subjects remained in the data set. Within- and between-reader (duplicate sets of 25 scans) and–observer (duplicate mean cIMT measurements in 5 subjects) variabilities were assessed twice a year [21]. Spearman correlation coefficients for intraobserver and intrareader measurements were > .95 and > .97, respectively, and mean differences (±2 SD) were < 1% (< 10%). Spearman correlation coefficient for between-observer and between-reader variabilities were > .90 and > .95, respectively. The mean differences (±2 SD) were < 5% (<15%). Prevalent carotid atherosclerosis was defined as cIMT above the sex- and age-related 75^th^ percentile or presence of atherosclerotic plaques.

### Statistics

Continuous data are expressed as median and 25^th^/75^th^ percentile. Nominal data are expressed as percentages. Differences between groups were calculated using Kruskal-Wallis (continuous variables) and χ^2^ test (nominal variables), respectively. First an analysis of variance with fixed effects for sex, age, smoking status, WHR and eGFR was fitted to ADMA, SDMA, and ARG tertiles (<33^rd^, 33^rd^ to 66^th^, >66^th^) to assess the quantitative associations between ARG derivatives and continuous log corrected cIMT (results are given as geometric mean and 95%-CI). In the second step three logistic regression models were used to analyze the association between ARG derivative serum concentrations and increased cIMT (> 75% sex- and age related percentile) as well as the presence of atherosclerotic plaques. For all calculations four different models were considered: univariate, partially adjusted for sex and age, fully adjusted for sex, age, smoking status, WHR and eGFR, and a risk factor model which included hypertension and diabetes in addition to the fully adjusted model. Odds ratios (OR) with 95% confidence intervals (CI) are shown for continuous serum ARG derivative concentrations and according to three groups based on the 33^rd^ and 66^th^ tertiles. The 33^rd^ to 66^th^ tertile was used as a reference in this analysis. Results for unadjusted and for models adjusted for sex, age, smoking status, WHR and eGFR are given. A *P* < 0.05 was considered statistically significant. All statistical analyses were performed in SAS 9.3 (SAS Institute Inc., Cary, NC, USA).

## Results

### General characteristics

In this data set 510 subjects, of 1999 total, had an increased cIMT. Subjects with increased cIMT were significantly less physically active and more often affected by hypertension and diabetes mellitus (Table 1). Furthermore, subjects with increased cIMT had significantly higher SDMA serum concentrations. No significant differences were observed for age, sex, smoking behavior, waist-to-hip ratio, estimated glomerular filtration rate, and serum concentrations of ADMA and ARG. In addition, the calculated ARG/ADMA ratio and DMA serum concentrations were also not significantly different between groups.

**Table 1:** General characteristics of the study population (median with 25^th^/75^th^ percentile).

		Subjects without increased IMT	Subjects with increased IMT	*P*
		(n = 1489)	(n = 510)	
Age (years)		61 (54; 69)	62 (54; 70)	0.11
% male		51.8	52.0	0.94
Smoking (%)	Ex-smoker	40.5	39.8	0.21
	Smoker	17.5	20.9	
	Nonsmoker	42.0	39.3	
**% Physically active**		**35.9**	**30.4**	**0.02**
WHR		0.89 (0.82; 0.95)	0.90 (0.83; 0.95)	0.15
eGFR (mL/min/1.73m2)		74 (66; 83)	74 (65; 82)	0.30
**Hypertensive (%)**		**65.4**	**78.4**	**< 0.001**
**Diabetes mellitus (%)**		**15.1**	**24.2**	**< 0.001**
**cIMT (mm)**		**0.83 (0.76; 0.93)**	**1.12 (0.99; 1.27)**	**< 0.001**
ADMA (μmol/L)		0.69 (0.61; 0.79)	0.70 (0.62; 0.79)	0.17
**SDMA (μmol/L)**		**0.47 (0.40; 0.55)**	**0.48 (0.41; 0.58)**	**0.05**
ARG (μmol/L)		150.1 (119.4; 184.6)	152.2 (122.3; 188.4)	0.34
DMA (μmol/L)		1.18 (1.04; 1.31)	1.19 (1.06; 1.36)	0.07
ARG/ADMA		218.5 (168.9; 266.5)	211.9 (170.9; 270.3)	0.81
**Plaque (%)**		**67.12**	**83.07**	**< 0.001**

Data are represented as median (interquartile range), or n (%). WHR indicates waist-to-hip ratio; eGFR, estimated-glomerular-filtration-rate; cIMT, carotid intima-media-thickness; ADMA, asymmetrical dimethylarginine; SDMA, symmetrical dimethylarginine; ARG, ˪-Arginine; DMA, dimethylarginine; ARG/ADMA, Arginine-asymmetrical dimethylarginine ratio.

### Multivariable ANOVA to assess the associations between continuous cIMT and ARG derivatives

**Fig 1:**
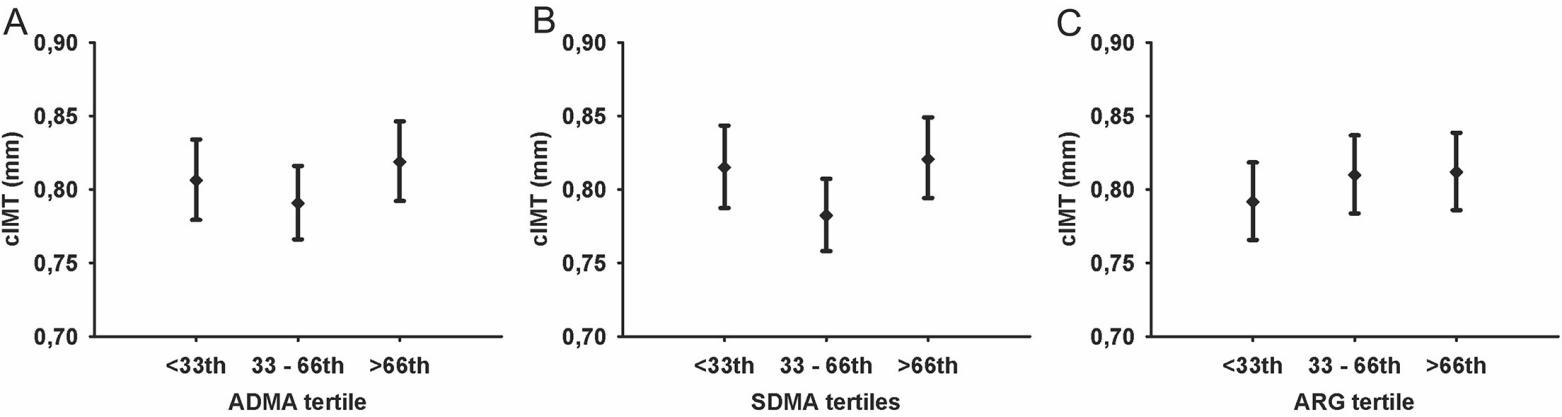
Estimated mean of cIMT with 95%-CI by ADMA (A), SDMA (B), and ARG (C). Multivariate analyses of variance were adjusted for age, sex, WHR, smoking, physical activity and eGFR. Post hoc comparison adjusted for multiple testing using Tukey. # *P* < 0.10 in post-hoc comparison. (cIMT indicates carotid intima-media thickness; ADMA, asymmetric dimethylarginine; SDMA, symmetric dimethylarginine; ARG, ˪-Arginine).

**Fig 2:**
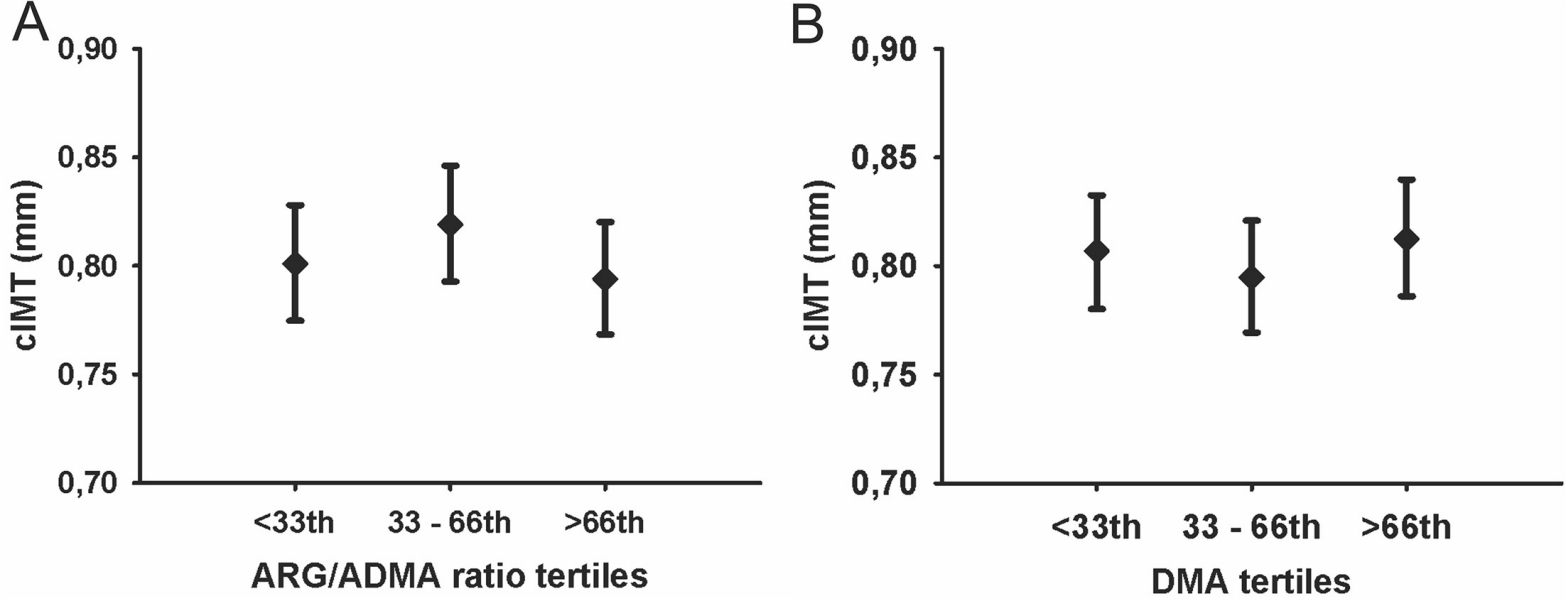
Estimated mean of cIMT with 95%-CI by ARG/ADMA ratio (A) and DMA (B). Multivariate analyses of variance were adjusted for age, sex, WHR, smoking, physical activity and GFR. Post-hoc comparison adjusted for multiple testing using Tukey. (cIMT indicates carotid intima-media thickness; ARG/ADMA, arginine asymmetrical dimethylarginine ratio; DMA, dimethylarginine).

In multivariable ANOVA with categorized ARG derivative serum SDMA concentration tertiles were related to cIMT (*P* = 0.07). Tukey adjusted post-hoc analysis identified higher cIMT in the group with high SDMA concentrations compared to the medium tertile (*P* < 0.10; Fig 1) [<33^rd^: 0.82 (95%-CI 0.79, 0.84) mm, 33^rd^–66^th^: 0.78 (95%-CI 0.76, 0.81) mm, >66^th^: 0.82 (95%-CI 0.79, 0.85) mm]. No significant differences in cIMT between groups were observed for tertiles of the ADMA and ARG [Fig 1; ADMA: <33^rd^: 0.81 (95%-CI 0.78, 0.83) mm; 33^rd^–66^th^: 0.79 (0.77, 0.82) mm; >66^th^: 0.82 (95%-CI 0.79, 0.84) mm; ARG: <33^rd^: 0.79 (95%-CI 0.77, 0.82) mm; 33^rd^–66^th^: 0.81 (95%-CI 0.78,0.84) mm; >66^th^: 0.81 (95%-CI 0.79; 0.84) mm] as well as the ARG/ADMA ratio and DMA [Fig 2; ARG/ADMA ratio: <33^rd^: 0.80 (95%-CI 0.77, 0.83) mm; 33^rd^–66^th^: 0.79 (95%-CI 0.77, 0.82) mm; >66^th^: 0.79 (95%-CI 0.77, 0.82) mm; DMA: <33^rd^: 0.81 (95%-CI 0.78, 0.83) mm; 33^rd^–66^th^: 0.79 (95%-CI 0.77, 0.82) mm; >66^th^: 0.81 (95%-CI 0.79, 0.84) mm].

### Logistic regression to analyze the associations between ARG derivatives and increased cIMT

In agreement with the ANOVA results, logistic regression analysis showed that high SDMA concentrations were associated with around 40% higher odds of increased cIMT in the unadjusted and fully adjusted model (Table 2). Higher DMA serum concentration was associated with a 70% increased odds of having increased cIMT in the unadjusted model (*P* = 0.02). Even though the significance was lost in the fully adjusted model, a strong trend (*P* = 0.06) for a positive association between serum DMA concentration and increased cIMT was still apparent. No significant associations were found for ADMA, ARG, and ARG/ADMA whether treated as continuous variables or based on tertiles. No differences between the partially and fully adjusted models were observed (S1 Table).

**Table 2:** Association of arginine derivatives with increased cIMT.

		OR (95%-CI) for increased IMT			
		Unadjusted	*P*	Fully adjusted	*P*
ADMA					
	cont.*	1.92 (0.92; 4.03)	0.08	1.74 (0.82; 3.70)	0.15
	categorized, ref: 33^rd^–66th				
	< 33^rd^	1.04 (0.81; 1.33)	0.78	1.04 (0.81; 1.34)	0.75
	> 66^th^	1.12 (0.94; 1.53)	0.14	1.18 (0.93; 1.51)	0.17
SDMA					
	**cont.** *	**2.62 (1.18; 5.84)**	**0.01**	**2.51 (0.99; 6.32)**	**0.05**
	categorized, ref: 33^rd^–66th				
	< 33^rd^	1.12 (0.87; 1.44)	0.37	1.12 (0.87; 1.44)	0.38
	**> 66** ^**th**^	**1.38 (1.08; 1.76)**	**0.01**	**1.39 (1.08; 1.79)**	**0.01**
ARG					
	cont. *	1.00 (0.99; 1.00)	0.35	1.00 (0.99; 1.00)	0.39
	categorized, ref: 33^rd^–66th				
	< 33^rd^	0.86 (0.67; 1.11)	0.25	0.86 (0.67; 1.10)	0.23
	> 66^th^	0.98 (0.77; 1.25)	0.85	0.95 (0.74; 1.21)	0.66
DMA					
	**cont.** *	**1.70 (1.09; 2.64)**	**0.02**	1.59 (0.99; 2.55)	0.06
	categorized, ref: 33rd–66th				
	< 33^rd^	1.07 (0.83; 1.37)	0.61	1.07 (0.83; 1.38)	0.60
	**> 66** ^**th**^	**1.28 (0.99; 1.63)**	**0.05**	1.26 (0.99; 1.62)	0.07
ARG/ADMA ratio					
	cont. *	1.00 (0.99; 1.00)	0.74	1.00 (0.99; 1.00)	0.67
	categorized, ref: 33rd–66th				
	< 33^rd^	0.96 (0.75; 1.22)	0.71	0.96 (0.75; 1.23)	0.74
	> 66^th^	0.90 (0.71; 1.15)	0.41	0.90 (0.71; 1.16)	0.42

OR = odds ratio, CI = confidence interval, adjusted for age, sex, smoking, WHR, and eGFR.

*OR for a 1 unit increase in serum ARG derivative concentration or ARG/ADMA ratio. cIMT indicates carotid intima-media thickness; WHR, waist-to-hip ratio; eGFR, estimated-glomerular-filtration-rate; ADMA, asymmetric dimethylarginine; SDMA, symmetric dimethylarginine; ARG, ˪-Arginine; DMA, dimethylarginine; ARG/ADMA, Arginine-asymmetrical dimethylarginine ratio.

### Logistic regression to analyze the association between ARG derivatives and the presence of atherosclerotic plaque

Logistic regression revealed a significantly positive association between continuous ADMA, SDMA or DMA serum concentrations and atherosclerotic plaque in the unadjusted analysis (Table 3). However, in the fully adjusted model all significances vanished. Using tertiles, regression analysis revealed that subjects with the highest ADMA levels had a 25% increased odds ratio for the presence of atherosclerotic plaque in the unadjusted model (*P* = 0.07). Furthermore, ARG in the highest tertile was significantly associated with an increased odds ratio of 31% and 41%, respectively, for the presence of atherosclerotic plaques in the unadjusted and adjusted model (Table 3). No significant association was identified for the presence of atherosclerotic plaques in the carotid artery with the ARG/ADMA ratio. No differences between the partially and fully adjusted models were observed (S2 Table).

**Table 3:** Association of arginine derivatives with the presence of atherosclerotic plaques.

		OR (95%-CI) for presence of atherosclerotic plaque			
		Unadjusted	*P*	Fully adjusted	*P*
ADMA					
	cont.*	**4.38 (2.06; 9.16)**	**< 0.01**	1.69 (0.72; 4.01)	0.23
	categorized, ref: 33rd- 66th				
	< 33rd	1.10 (0.87; 1.40)	0.41	1.11 (0.85; 1.45)	0.45
	> 66th	1.25 (0.99; 1.58)	0.07	1.21 (0.92; 1.58)	0.17
SDMA					
	cont. *	**7.80 (3.30; 18.41)**	**< 0.01**	0.93 (0.31; 2.74)	0.89
	categorized, ref: 33rd- 66th				
	< 33rd	1.07 (0.84; 1.36)	0.59	1.05 (0.80; 1.38)	0.74
	> 66th	0.93 (0.74; 1.17)	0.54	0.94 (0.72; 1.26)	0.66
ARG					
	cont. *	1.00 (0.99; 1.00)	0.58	1.00 (0.99; 1.00)	0.30
	categorized, ref: 33rd- 66th				
	< 33rd	1.09 (0.86; 1.37)	0.50	1.15 (0.87; 1.51)	0.51
	**> 66th**	**1.31 (1.03; 1.66)**	**0.03**	**1.41 (1.07; 1.85)**	**0.01**
DMA					
	cont. *	**3.14 (1.98; 4.97)**	**< 0.01**	1.21 (0.70: 2.09)	0.50
	Categorized, ref: 33rd- 66th				
	< 33rd	0.99 (0.78; 1.25)	0.90	1.00 (0.76; 1.32)	0.99
	> 66th	0.98 (0.77; 1.24)	0.85	0.97 (0.74; 1.28)	0.85
ARG/ADMA ratio					
	cont. *	0.99 (0.99; 1.00)	0.09	1.00 (0.99; 1.00)	0.86
	Categorized, ref: 33rd- 66th				
	< 33rd	0.95 (0.75; 1.21)	0.69	0.96 (0.73; 1.27)	0.57
	> 66th	0.97 (0.76; 1.23)	0.78	1.01 (0.77; 1.33)	0.67

OR = odds ratio, CI = confidence interval, adjusted for age, sex, smoking, WHR, and GFR.

*OR for a 1 unit increase in serum ARG derivative concentration or ARG/ADMA ratio. cIMT indicates carotid intima-media thickness; WHR, waist-to-hip ratio; eGFR, estimated-glomerular-filtration-rate; ADMA, asymmetric dimethylarginine; SDMA, symmetric dimethylarginine; ARG, ˪-Arginine; DMA, dimethylarginine; ARG/ADMA, Arginine-asymmetrical dimethylarginine ratio.

## Discussion

The main result of this study is that serum concentrations of ARG derivatives were independently related with atherosclerosis in a large population-based adult cohort from Northeast Germany. Specifically, high serum SDMA and DMA concentrations were positively associated with cIMT after correction for sex, age, smoking, waist-to-hip ratio, and estimated glomerular filtration rate. This association was not influenced by cardiovascular risk factors like hypertension and diabetes (S1 Table). Furthermore, increased serum ARG concentration was significantly positively associated with the presence of atherosclerotic plaque in the extracranial carotid arteries. Epidemiological studies have previously reported associations between markers of subclinical atherosclerosis with ARG derivatives [22,23]. However, whether this association is true for all ARG derivatives across different populations was so far unknown.

The importance of ARG is determined by its pivotal role in NO signaling. Specifically, ARG is the natural precursor of the atheroprotective gas NO [2]. In a large population-based cohort from Peru, serum ARG concentration independently predicted markers of subclinical CVD [24]. Specifically, a positive association between ARG and systolic hypertension, higher central blood pressure, and lower total artery compliance was reported. In contrast, in a Chinese population significantly lower ARG concentrations were found in hypertensive subjects compared to normotensive controls [25]. However, no significant differences were identified when normotensive and hypertensive patients with diabetes mellitus were compared [26]. A Turkish study reported a significantly inverse correlation between ARG and cIMT in patients with cardiac syndrome X [27]. Interestingly, our results show a heterogeneous relationship of ARG with atherosclerosis in the extracranial carotid artery. While there was no significant association of ARG serum concentration with cIMT, a positive relation for the presence of atherosclerotic plaque was revealed after adjustment for relevant determinants of atherosclerosis. High ARG concentrations were independently associated with an increased risk for the presence of atherosclerotic plaques by nearly 41% (Table 3). While our results may seem contradictory to the well-established anti-atherosclerotic NO-dependent effects of ARG, one may propose that due to the age of the investigated population (> 45 years) oxidative stress in endothelial cells was high. This could cause decreased NO bioavailability which may have led to a compensatory upregulation of ARG. However, in combination with results from previous studies [24,25,27], our results mostly indicate that whether ARG concentration can be used as a surrogate marker for atherosclerotic disease progression depends not just on the health status of the population of interest, but also on how atherosclerosis is defined and assessed.

ADMA competes for ARG binding sites on eNOS thereby directly inhibiting its actions and reducing NO bioavailability [10]. A large meta-analysis reported that circulating ADMA concentration positively correlates with cIMT [11]. Our results cannot confirm a significant association of serum ADMA concentration with cIMT or with presence of atherosclerotic plaques (Fig 1; Tables 2 and 3). Interestingly, the median serum ADMA concentration across the SHIP participants in this study was 0.69 and 0.70 μmol/L for subjects without and with increased cIMT, respectively. Considering that the median serum concentration in a reference data set of healthy SHIP-0 participants (no diabetes mellitus, cardiovascular disease, increased blood pressure, chronic kidney disease and increased BMI) was 0.64 μmol/L (2.5^th^ and 97.5^th^ percentile 0.41 μmol/L and 0.95 μmol/L) [28], the ADMA serum concentration in this data set is rather high. This may mask a potential correlation due to a ceiling effect. Furthermore, a second potential reason for this discrepancy is that the majority of previous studies used patients instead of population-based cohorts [8,9,29]. Therefore, differences in established behavioral and biological risk factors of atherosclerosis may influence the suitability of ADMA as an atherosclerotic biomarker in the general population. Nonetheless, two reports from population-based cohorts support our findings of no significant association between ADMA concentration and common cIMT [30,31]. However, two studies reported a significantly positive association between circulating ADMA concentrations and cIMT. These studies investigated cohorts in Japan and Peru [24,32]. One may speculate that the different results could be explained by the origin of the cohorts since geographic differences in CVD susceptibility are well established [33]. Furthermore, ethnicity specific ADMA reference concentrations have previously been reported for African-Americans, mixed non-Hispanics and Whites [34]. This concept of regional heterogeneity and ethnicity may influence the suitability of ADMA as a biomarker for atherosclerosis. This is supported by studies conducted in healthy children. Specifically, one study reported a significant correlation between ADMA concentration and cIMT in Australian children [35]. However, this association was not significant for a study conducted in Polish children [36]. Therefore, whether circulating ADMA levels are a biomarker for atherosclerosis may be dependent upon the geographical location and ethnicity of the population of interest.

SDMA is a structural isomer of ADMA and inhibits cationic amino acid transporters which are essential for the transcellular membrane passage of ARG [10]. In a cohort of patients with dilated cardiomyopathy SDMA predicted all-cause mortality [29]. In a group of patients with stable coronary artery disease SDMA was related to an increased number of CVD events [8]. Likewise, in population-based cohorts SDMA concentration has been identified as an independent predictor of all-cause and cardiovascular mortality [22,23]. However, whether SDMA predicts atherosclerotic disease progression in the general population is currently unclear. In our large population-based adult cohort from Northeast Germany, high levels of serum SDMA concentration related to a 39% increased odds ratio for high cIMT. Interestingly, SDMA concentration was not significantly associated with the presence of atherosclerotic plaque in the extracranial carotid arteries. Very few previous population-based cohorts investigated the potential relation between SDMA concentrations and atherosclerosis. In the Dallas Heart Study SDMA was associated with coronary artery calcium and aortic wall thickness [22]. In an older subject population (mean age 76 years), SDMA concentration was positively correlated with increased cIMT and the presence of atherosclerotic plaques [13]. Overall, the current data does not allow for a definite conclusion on whether SDMA concentration is related with atherosclerosis in the general population.

In order to enable further interpretation of our results we calculated the ratio of ARG and its inhibitor ADMA as well as DMA. The ARG/ADMA ratio has previously been identified as an independent predictor of mortality in patients with dilated cardiomyopathy [29]. In addition, a positive correlation between ARG/ADMA and all-cause mortality was identified in a 10 year follow-up investigation of the Framingham Heart Study [31]. Furthermore, in patients with cardiac syndrome X the ARG/ADMA ratio was inversely associated with cIMT [27]. Moreover, in a Japanese population the ARG/ADMA ratio with significantly associated with IMT [37]. Among SHIP participants the ARG/ADMA ratio did not relate with either increased cIMT or presence of atherosclerotic plaques (Fig 2; Tables 2 and 3). Interestingly, DMA was positively associated with increased cIMT, while no association with the presence of atherosclerotic plaque was observed in the adjusted analysis. Overall, ARG/ADMA ratio and DMA concentration may correlate with some markers of subclinical atherosclerosis, but this will be depending upon the choice of definition of asymptomatic CVD. Specifically, one may speculate that cIMT and presence of atherosclerotic plaque are surrogate markers for different disease stages. While cIMT may represent a marker of earlier phases of atherosclerotic disease, plaques are present during later stages. Thus, the strength of a potential association with ARG derivatives might be different for cIMT and plaques, possibly hampering its detection in a population-based setting. We have observed similar differences also in previous analyses when we investigated the relation between thyroid function or total serum testosterone levels with cIMT and prevalent carotid atherosclerotic plaques among large samples from SHIP [38–40].

We acknowledge several limitations in our analysis. Most importantly, our cross-sectional results do not imply an underlying biological mechanism. In addition, we recognize that we did not exclude subjects with previous CVD events like stroke or myocardial infarction. Further, while antihypertensive and lipid-lowering medication may influence carotid atherosclerosis and cIMT [41,42], their effect is time dependent. Unfortunately, no information about the duration of the treatment was available. Thus, we cannot completely exclude that this might have affected our findings. This is due to the fact that we aimed to analyze an older general population cohort.

In summary, this is the most comprehensive epidemiological analysis correlating diverse ARG derivatives with two distinct pathophysiological markers of atherosclerotic disease progression. The results of this study show that while serum concentrations of ARG and SDMA are positively associated with atherosclerosis, no correlation was found for ADMA. Furthermore, the ARG/ADMA ratio was not associated with either increased cIMT or presence of atherosclerotic plaques. However, high DMA serum concentration significantly increased the odds for the presence of atherosclerotic plaques in our cohort. Therefore, whether ARG derivatives are atherosclerotic biomarkers deserves further research.

## Supporting Information

S1 Table Association of arginine derivatives with increased cIMT. OR = odds ratio, CI = confidence interval, *OR for a 1 unit increase in serum ARG derivative concentration or ARG/ADMA ratio. cIMT indicates carotid intima-media thickness; WHR, waist-to-hip ratio; eGFR, estimated-glomerular-filtration-rate; ADMA, asymmetric dimethylarginine; SDMA, symmetric dimethylarginine; ARG, ˪-Arginine; DMA, dimethylarginine; ARG/ADMA, Arginine-asymmetrical dimethylarginine ratio.
(PDF)

S2 Table Association of arginine derivatives with the presence of atherosclerotic plaques. OR = odds ratio, CI = confidence interval, *OR for a 1 unit increase in serum ARG derivative concentration or ARG/ADMA ratio. cIMT indicates carotid intima-media thickness; WHR, waist-to-hip ratio; eGFR, estimated-glomerular-filtration-rate; ADMA, asymmetric dimethylarginine; SDMA, symmetric dimethylarginine; ARG, ˪-Arginine; DMA, dimethylarginine; ARG/ADMA, Arginine-asymmetrical dimethylarginine ratio.
(PDF)
