# Supplementary material for: L-Arginine and SDMA Serum Concentrations Are Associated with Subclinical Atherosclerosis in the Study of Health in Pomerania (SHIP)
Source: PLoS One. 2015 Jun 22;10(6):e0131293. doi: 10.1371/journal.pone.0131293 (PMC4476678; doi:10.1371/journal.pone.0131293)
Supplement: S1 Table — OR = odds ratio, CI = confidence interval, *OR for a 1 unit increase in serum ARG derivative concentration or ARG/ADMA ratio. cIMT indicates carotid intima-media thickness; WHR, waist-to-hip ratio; eGFR, estimated-glomerular-filtration-rate; ADMA, asymmetric dimethylarginine; SDMA, symmetric dimethylarginine; ARG, ˪-Arginine; DMA, dimethylarginine; ARG/ADMA, Arginine-asymmetrical dimethylarginine ratio. (PDF) [file pone.0131293.s001.pdf]

| | | OR (95% CI) for increased IMT | | | | | | | |
|---|---|---|---|---|---|---|---|---|---|
| | | Unadjusted | *P* | Adjusted for sex and age | *P* | Adjusted for age, sex, eGFR, smoking, WHR | *P* | Adjusted for age, sex, eGFR, smoking, WHR, diabetes, hypertension | *P* |
| ADMA | | | | | | | | | |
| | cont.* | 1.92 (0.92; 4.03) | 0.08 | 1.79 (0.85; 3.77) | 0.13 | 1.74 (0.82; 3.70) | 0.15 | 1.76 (0.82; 3.80) | 0.14 |
| | categorized, ref: 33rd - 66th | | | | | | | | |
| | < 33rd | 1.04 (0.81; 1.33) | 0.78 | 1.04 (0.81; 1.33) | 0.78 | 1.04 (0.81; 1.34) | 0.75 | 1.10 (0.90; 1.48) | 0.94 |
| | > 66th | 1.12 (0.94; 1.53) | 0.14 | 1.20 (0.94; 1.52) | 0.15 | 1.18 (0.93; 1.51) | 0.17 | 1.15 (0.90; 1.50) | 0.26 |
| SDMA | | | | | | | | | |
| | **cont.*** | **2.62 (1.18; 5.84)** | **0.01** | **2.34 (1.01; 5.43)** | **0.05** | **2.51 (0.99; 6.32)** | **0.05** | **2.84 (1.11; 7.25)** | **0.03** |
| | categorized, ref: 33rd - 66th | | | | | | | | |
| | < 33rd | 1.12 (0.87; 1.44) | 0.37 | 1.12 (0.87; 1.44) | 0.39 | 1.12 (0.87; 1.44) | 0.38 | 1.05 (0.81; 1.36) | 0.71 |
| | **> 66th** | **1.38 (1.08; 1.76)** | **0.01** | **1.38 (1.08; 1.76)** | **0.01** | **1.39 (1.08; 1.79)** | **0.01** | **1.36 (1.05; 1.75)** | **0.02** |
| ARG | | | | | | | | | |
| | cont.* | 1.00 (0.99; 1.00) | 0.35 | 1.00 (0.99; 1.00) | 0.32 | 1.00 (0.99; 1.00) | 0.39 | 1.00 (0.99; 1.00) | 0.44 |
| | categorized, ref: 33rd - 66th | | | | | | | | |
| | < 33rd | 0.86 (0.67; 1.11) | 0.25 | 0.85 (0.67; 1.10) | 0.21 | 0.86 (0.67; 1.10) | 0.23 | 0.84 (0.65; 1.08) | 0.34 |
| | > 66th | 0.98 (0.77; 1.25) | 0.85 | 0.96 (0.75; 1.23) | 0.76 | 0.95 (0.74; 1.21) | 0.66 | 0.92 (0.72; 1.18) | 0.17 |
| DMA | | | | | | | | | |
| | **cont.*** | **1.70 (1.09; 2.64)** | **0.02** | **1.60 (1.01; 2.52)** | **0.05** | 1.59 (0.99; 2.55) | 0.06 | **1.65 (1.02; 2.66)** | **0.04** |
| | categorized, ref: 33rd – 66th | | | | | | | | |
| | < 33rd | 1.07 (0.83; 1.37) | 0.61 | 1.07 (0.83; 1.37) | 0.61 | 1.07 (0.83; 1.38) | 0.60 | 1.07 (0.83; 1.39) | 0.59 |
| | **> 66th** | **1.28 (0.99; 1.63)** | **0.05** | **1.28 (1.01; 1.64)** | **0.05** | 1.26 (0.99; 1.62) | 0.07 | **1.29 (1.00; 1.66)** | **0.05** |
| ARG/ADMA rat | | | | | | | | | |
| | cont.* | 1.00 (0.99; 1.00) | 0.74 | 1.00 (0.99; 1.00) | 0.62 | 1.00 (0.99; 1.00) | 0.67 | 1.00 (0.99; 1.00) | 0.69 |
| | categorized, ref: 33rd – 66th | | | | | | | | |
| | < 33rd | 0.96 (0.75; 1.22) | 0.71 | 0.95 (0.74; 1.22) | 0.68 | 0.96 (0.75; 1.23) | 0.74 | 0.93 (0.73; 1.20) | 0.6 |
| | > 66th | 0.90 (0.71; 1.15) | 0.41 | 0.90 (0.70; 1.15) | 0.40 | 0.90 (0.71; 1.16) | 0.42 | 0.89 (0.70; 1.14) | 0.37 |
