# Supplementary material for: L-Arginine and SDMA Serum Concentrations Are Associated with Subclinical Atherosclerosis in the Study of Health in Pomerania (SHIP)
Source: PLoS One. 2015 Jun 22;10(6):e0131293. doi: 10.1371/journal.pone.0131293 (PMC4476678; doi:10.1371/journal.pone.0131293)
Supplement: S2 Table — OR = odds ratio, CI = confidence interval, *OR for a 1 unit increase in serum ARG derivative concentration or ARG/ADMA ratio. cIMT indicates carotid intima-media thickness; WHR, waist-to-hip ratio; eGFR, estimated-glomerular-filtration-rate; ADMA, asymmetric dimethylarginine; SDMA, symmetric dimethylarginine; ARG, ˪-Arginine; DMA, dimethylarginine; ARG/ADMA, Arginine-asymmetrical dimethylarginine ratio. (PDF) [file pone.0131293.s002.pdf]

		OR (95% CI) for presence of atherosclerotic plaque							
		Unadjusted	P	Adjusted for sex and age	P	Adjusted for age, sex, eGFR, smoking, WHR	P	Adjusted for age, sex, eGFR, smoking, WHR, diabetes, hypertension	P
ADMA									
	**cont.***	**4.38 (2.06; 9.16)**	**< 0.01**	1.69 (0.72; 3.95)	0.23	1.69 (0.72; 4.01)	0.23	1.69 (0.70; 4.10)	0.24
	categorized, ref: 33rd - 66th								
	< 33rd	1.10 (0.87; 1.40)	0.41	1.08 (0.83; 1.41)	0.57	1.11 (0.85; 1.45)	0.45	1.10 (0.84; 1.44)	0.51
	> 66th	1.25 (0.99; 1.58)	0.07	1.20 (0.92; 1.56)	0.19	1.21 (0.92; 1.58)	0.17	1.19 (0.91; 1.56)	0.21
SDMA									
	**cont.***	**7.80 (3.30; 18.41)**	**< 0.01**	**0.40 (0.15; 1.11)**	**0.08**	0.93 (0.31; 2.74)	0.89	1.00 (0.33; 3.01)	0.99
	categorized, ref: 33rd - 66th								
	< 33rd	1.07 (0.84; 1.36)	0.59	1.10 (0.84; 1.44)	0.51	1.05 (0.80; 1.38)	0.74	0.99 (0.75; 1.31)	0.93
	> 66th	0.93 (0.74; 1.17)	0.54	0.83 (0.64; 1.09)	0.18	0.94 (0.72; 1.26)	0.66	0.91 (0.69; 1.20)	0.51
ARG									
	cont.*	1.00 (0.99; 1.00)	0.58	1.00 (0.99; 1.00)	0.19	1.00 (0.99; 1.00)	0.30	1.00 (0.99; 1.00)	0.33
	categorized, ref: 33rd - 66th								
	< 33rd	1.09 (0.86; 1.37)	0.50	1.11 (0.85; 1.45)	0.45	1.15 (0.87; 1.51)	0.51	1.15 (0.87; 1.51)	0.33
	**> 66th**	**1.31 (1.03; 1.66)**	**0.03**	**1.41 (1.08; 1.85)**	**0.01**	**1.41 (1.07; 1.85)**	**0.01**	**1.41 (1.07; 1.85)**	**0.02**
DMA									
	**cont.***	**3.14 (1.98; 4.97)**	**< 0.01**	0.97 (0.57; 1.66)	0.90	1.21 (0.70: 2.09)	0.50	1.24 (0.71; 2.17)	0.45
	Categorized, ref: 33rd - 66th								
	< 33rd	0.99 (0.78; 1.25)	0.90	1.01 (0.77; 1.33)	0.92	1.00 (0.76; 1.32)	0.99	1.01 (0.77; 1.34)	0.92
	> 66th	0.98 (0.77; 1.24)	0.85	0.91 (0.70; 1.20)	0.51	0.97 (0.74; 1.28)	0.85	1.00 (0.76; 1.31)	0.97
ARG/ADMA rat									
	cont.*	0.99 (0.99; 1.00)	0.09	1.00 (0.99; 1.00)	0.72	1.00 (0.99; 1.00)	0.86	1.00 (0.99; 1.00)	0.88
	Categorized, ref: 33rd - 66th								
	< 33rd	0.95 (0.75; 1.21)	0.69	0.93 (0.71; 1.22)	0.59	0.96 (0.73; 1.27)	0.57	0.95 (0.72; 1.26)	0.74
	> 66th	0.97 (0.76; 1.23)	0.78	0.99 (0.76; 1.30)	0.95	1.01 (0.77; 1.33)	0.67	1.01 (0.77; 1.34)	0.92
